# How Different Are Industrial, Artisanal and Homemade Soft Breads?

**DOI:** 10.3390/foods11101484

**Published:** 2022-05-19

**Authors:** Bastien Maurice, Anne Saint-Eve, Aurélia Pernin, Pascal Leroy, Isabelle Souchon

**Affiliations:** 1 Université Paris-Saclay, INRAE, AgroParisTech, UMR SayFood, F-91120 Palaiseau, France; bastien.maurice@inrae.fr (B.M.); anne.saint-eve@inrae.fr (A.S.-E.); aurelia.pernin@agroparistech.fr (A.P.); 2 Université Paris-Saclay, INRAE, UMR PSAE, F-91120 Palaiseau, France; pascal.leroy@inrae.fr; 3 INRAE, Avignon Université, UMR SQPOV, F-84000 Avignon, France

**Keywords:** multicriteria mapping, processed food, texture, volatile, GC-MS, sandwich bread, white bread, oxidation, fermentation, aroma

## Abstract

Soft bread has a significant relevance in modern diets, and its nutritional impact on human health can be substantial. Within this product category, there is an extensive range of ingredients, formulations, and processing methods, which all contribute to the vast diversity found in the final products. This work compared the impact of three different processing methods (industrial, artisanal, and homemade preparation) on the technological (formulation and processing, as they are interconnected in real-life conditions), nutritional, and physicochemical properties of soft bread. In total, 24 types of soft bread were analyzed: 10 industrial, 6 artisanal, and 8 homemade. Although production diagrams were similar among the three methods, industrial recipes contained on average more ingredients and more additives. Industrial bread was lower in saturated fat compared to the other two groups, but contained more sugar than homemade bread. The physical properties of all loaves were comparable, with the exception of higher crumb elasticity in industrial bread compared to homemade. An analysis of volatile molecules revealed more lipid oxidation markers in industrial bread, more fermentation markers in artisanal bread, and fewer markers of Maillard reactions in homemade bread. Chemical reactions during processing seem to be the principal criterion making possible to discriminate the different processing methods. These results offer a quantitative assessment of the differences within a single product category, reflecting the real-world choices for consumers.

## 1. Introduction

In recent centuries, food has become increasingly industrialized [1]. This process has led to the development of numerous manufacturing operations and novel formulations, all with the aim of offering consumers around the world a diverse range of food products that meet the quality standards and norms of globalized trade [2,3].

The magnitude of this transformation has fueled questions about its potential impacts on human health [4]. In particular, food processing has come under increased scrutiny, to the point that food classification schemes have been developed that are-based primarily on the processing and formulation levels (where, by whom, why and how foods were prepared) [5], such as EPIC (European Prospective Investigation into Cancer and Nutrition) [6] and NOVA (a name, not an acronym) [7]. However, these classification schemes have often been criticized because they integrate multiple dimensions linked to the processing and formulation [8] and because they suffer from a lack of robustness [9,10]. A first step in clarifying the existing ambiguity would be to distinguish the formulation of a food (i.e., its recipe) from its processing (i.e., unit operations), as these concepts are sometimes perceived differently in the field of public health from in the realm of food science [11]. Although it is clear that the consumption of ultra-processed food is linked with negative health consequences, the hypotheses proposed to explain this effect have been numerous and varied.

Some explanations target the presence of additives or contaminants originating from contact materials and packaging, while others highlight the formation of certain compounds during processing [4]. Modifications of the food matrix in such a way as to increase the rate of ingestion and kinetics of digestion have also been proposed as a possible hypothesis [12]. Other hypotheses lay blame on the extreme palatability of ultra-processed foods, with complex formulations, often using flavoring agents [4].

Formulation and processing are both essential in determining the structure of a finished product, which is a key element in shaping both sensory properties and nutritional characteristics. For example, the density of bread is known to influence its glycemic index [13]. Formulation and processing are also responsible for the development of aroma compounds; for instance, in bread, flour type, fermentation and baking conditions influence the production of volatile compounds [14,15]. Furthermore, the association between formulation and processing can, via Maillard reactions, create molecules such as acrylamide, 5-hydroxymethylfurfural, and furan, which all contribute to the toxicological risk posed by the final product and its potentially harmful effects on health [16]. Furans appear in thermally processed foods, and levels of furan in toasted bread were found to be correlated with browning [17], and to levels of furfural in sponge cakes [18]. A relationship was also identified between thermal processing and levels of furfural, 5-hydroxymethylfurfural, and certain color parameters, but not acrylamide [19].

With increasing awareness of the impact of food on human health and the environment, consumers have become more attracted to homemade and local products, which convey a more virtuous image of being less processed and more authentic [20]. In consumer perceptions, there is a well-established duality between homemade products and mass-produced industrial goods. Between these two lies artisanal production, which is associated with the traditional aspects of homemade production but with higher volumes, and without the extensive automation and mass production found in industrial settings [21]. However, the distinctions between these three processing methods are not clearly or objectively established, and it is difficult to predict how differences in formulation and processing among the three methods may contribute to variation in the properties of the final food product.

To date, only a few studies have attempted to compare the characteristics of products within a single food category based on different methods of food processing (i.e., industrial, artisanal, and homemade). In the case of bread, the milling of wheat and the breadmaking process have been investigated for their effects on the nutritional quality of bread [22]. The fermentation step, in particular the use of sourdough or industrial yeast, has also been the subject of particular attention [23,24]. Different cooking methods—specifically, steaming, baking, and toasting—have been found to have an impact on bread quality and on the formation of volatile compounds from Maillard reactions [14,25,26,27]. Comparisons of industrial and traditional French baguettes have highlighted differences in structure and texture [28], as well as in aroma profiles [29]. Overall, though, little consideration has been given to the sometimes extreme variability that can exist among products within this category.

Therefore, we chose to focus on plain soft bread as a processed food model because it is a mass-marketed food product that has been a dominant element of the traditional diet of many countries and is largely consumed worldwide [30]. Indeed, bakery products account for a large part of the ultra-processed foods consumed by Europeans [31]. For instance, data from 2017 indicate that nearly 90% of the French population consumes refined bread and dry bakery products, with an average of more than 100 g per person per day [32]. As a dietary staple, the influence of bread on health should not be discounted, especially considering that different methods of processing—from the grain to the finished loaf—are known to influence the nutritional quality of the final product [33]. Previous studies have examined the effect of different processing methods on a single recipe, but the novelty of the present study is that it takes into account the actual variability in products available to consumers. Our goal was to analyze the real-world diversity that can be found in both recipes and processing techniques, as demonstrated by the range of industrial examples on shelves and the myriad recipes found online or from artisanal bakeries.

Overall, the objective of this study was to compare three different methods of processing (industrial, artisanal, and homemade) and their impacts on the technological, nutritional, and physicochemical properties of soft bread. To that end, within each method of preparation, we examined numerous properties of several different breads. Specifically, we assessed the number of ingredients and additives in recipes, an objective indicator of food transformation, amounts of macronutrients and salt, a global nutritional score, color, density, rheological properties, and volatile molecule profiles. By analyzing multiple types of bread within each processing method, we aimed to gain a better understanding of the broad diversity within this food category.

## 2. Materials and Methods

### 2.1. Industrial, Artisanal, and Homemade Soft Bread

To compare the three different methods of processing—industrial, artisanal, and homemade—we selected and obtained different examples of soft bread from each group.

First, we searched the Open Food Facts database (https://fr.openfoodfacts.org, accessed on 1 April 2021) for all items corresponding to industrially prepared plain soft bread in France; this search returned 109 products (gluten-free and toasts excluded). By means of a hierarchical ascending classification (data not shown), we selected 10 industrial soft breads, which were representative of the variety within this group with respect to nutritional characteristics and the number of ingredients and additives. Six types of artisanal soft bread were purchased in different local bakeries (Yvelines area, France), and eight types of homemade bread were prepared from popular online recipes. Ingredients and recipes for the 24 types of soft bread examined in this study are listed in Table 1.

To take into account intra-product variability, we sampled three separate loaves of each of the 24 types of bread for the different characterizations described below. All sampling was conducted on fresh bread, i.e., at least one week before the date of minimum durability (“best before” date) on the packaging and less than 5 h after opening for industrial loaves, less than 5 h after purchase for artisanal loaves, and less than 5 h after baking for homemade loaves. Samples were frozen at −80 °C and transported at a temperature lower than 5 °C for the determination of nutritional values.

### 2.2. Recipes and Technological Data

With the exception of additives, every item on the labeled recipe was counted as an ingredient. Additives were counted separately, labeled with either their common names (e.g., ascorbic acid) or E numbers (e.g., E300). Missing proportions of ingredients were determined using Anatole^©^ software [34], which analyzes the mass balances of different nutrients using the recipe and nutritional values on the label, in light of labeling regulations.

The degree of processing was evaluated using the NOVA classification (Table 1) and its four processing categories (i.e., NOVA1: unprocessed or minimally processed foods; NOVA2: processed culinary ingredients; NOVA3: processed foods; NOVA4: ultra-processed foods) [7].

**Figure 1:**
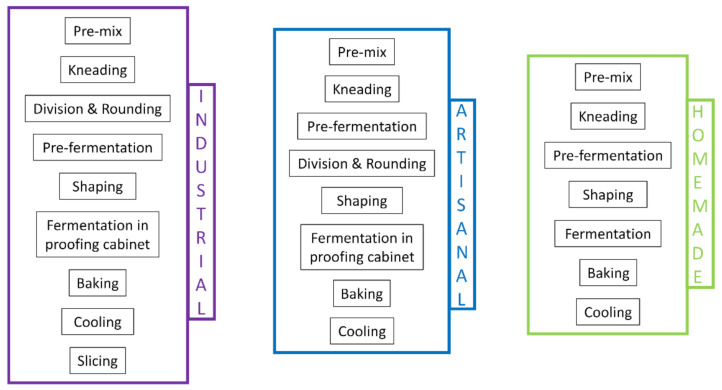
Examples of a production diagram for industrial, artisanal, and homemade soft breads, listing the general unit operations for each.

To dissociate the effects of formulation from those of food processing, and to better account for the degree of transformation of each bread, we developed an algorithm based on processing diagrams and recipes. Each unit operation of the processing diagram was associated with a score that reflects the time and intensity of the process and its impact (chemical, physical, or biological) on the food product: the more extreme the process conditions (temperature, pressure, time), the higher the impact and thus the higher the score (Table S1a). In this way, a Process-Score was calculated for each ingredient (Table S1b) according to the unit operations in its processing diagram. An example of a production diagram for soft bread is shown in Figure 1.

### 2.3. Nutritional Data

The nutritional values (per 100 g: energy density, total carbohydrates, sugars, dietary fibers, proteins, total fats, saturated fatty acids, and salt) of bread were experimentally determined by an accredited laboratory (Eurofins Scientific, Nantes, France), according to the legal labeling requirements in EU regulation 1169/2011. This was performed for five industrial, six artisanal, and eight homemade breads. For the 13 breads with both labeled and experimental nutritional values, the only deviations from labeled values were for carbohydrate, sugar, and salt content (*p* = 0.001, 0.006, and 0.002, respectively), for which mean differences of 8.9%, −14.0%, and 12.4%, respectively, were observed between the experimental and labeled values. However, such deviations are still within the legal tolerance laid out by regulation 1169/2011. Therefore, for the five industrial soft breads that were not experimentally tested, the nutritional data from the label were used. We also calculated Rayner’s score and the Nutri-Score as described by the French Public Health Agency [35]. 

### 2.4. Characterization of the Physical Properties of Soft Bread

All the following analyses were performed in triplicate.

#### 2.4.1. Soft Bread Density

The apparent density of each bread was evaluated by first weighing three slices from the middle (away from the ends) of the loaf using a 0.01-g-precision scale (XT 6200C, Precisa, Dietikon, Switzerland) and then measuring the volume of the slices using image analysis of pictures taken under controlled light conditions (ScanCube, Altawak Technologies, Paris, France). 

#### 2.4.2. Colorimetry

For the crumbs of each bread, we evaluated the color parameters L* (scale of 0 (dark) to 100 (light)), a* (scale of −128 (green) to 127 (red)), and b* (scale of −128 (blue) to 127 (yellow)) using a spectrocolorimeter (CM-2600d, Minolta, Tokyo, Japan) in SCE mode (Specular Component Excluded, i.e., excluding surface conditions). Chromaticity, C* (scale of dull to bright), was calculated as $C^{*}\;=\;\sqrt{a^{*2}\;+\;b^{*2}}$.

#### 2.4.3. Texture Properties

Uniaxial compression–relaxation tests were performed using a texture analyzer (TA.XT plus, Stable Micro Systems, Surrey, UK) at room temperature (20 °C). A 30-mm-diameter flat circular probe was applied to 30-mm-diameter samples taken from the center of a bread slice, employing a constant speed of 0.5 mm·s^−1^ and a strain rate of 40%, followed by a holding time of 20 s. Values of Young’s modulus (F_max_) and the percentage of relaxation were calculated, respectively, as the values of the initial slope of the stress/strain curve under maximum applied force, and the difference between the maximum applied force and the force after 15 s of relaxation).

#### 2.4.4. Physical Determination of Water Content

Bread moisture content were measured by gravimetric method at 110 °C until a constant weight was achieved, consistent with Mathieu et al. (2016) [36].

### 2.5. Characterization of the Chemical Properties of Soft Bread

#### 2.5.1. Analysis of Volatile Compounds

To compare the profiles of volatile compounds among the 24 soft breads, 120 g of a sample of 20% (weight/weight) bread crumb and 80% (weight/weight) water were blended for 90 s with an ULTRA-TURRAX^®^ device (T25, IKA-Werke, Staufen im Breisgau, Germany) at 8000 rpm (rotations per minute) in a beaker surrounded by ice. From this, 5 g were placed in a vial and kept frozen at −80 °C. Samples were placed at 4 °C 16 h prior to Dynamic Headspace System coupled with GC-MS (Gas Chromatography–Mass Spectrophotometry) analysis, described elsewhere [37]. The chromatogram was recorded, and the areas of the chromatographic peaks were integrated using MassHunter software (Agilent Technologies, Santa Clara, CA, USA) from the total ion current (TIC), and, in cases of co-elution, using an extracted ion chromatogram (EIC) (the selected ions for each compound treated by EIC are available in Table S2). Compounds were identified by comparison of their mass spectra with those in the NIST (National Institute of Standards and Technology) 2017 Mass Spectral Library, and verification was performed using their Kovats indexes. The compounds that could be used to successfully discriminate among processing methods (industrial, artisanal, or homemade) are presented in Table 2 (46 out of 81). For statistical analysis, the peaks below the limit of detection (i.e., three times the noise of the baseline signal) were set at 1000 for TIC and 100 for EIC.

#### 2.5.2. Quantification of Key Volatile Compounds

On a subset of 11 soft breads, we used the standard addition method to determine the concentrations of six target volatile molecules. These compounds were selected from the scientific literature as markers of specific processes involved in lipid oxidation (2-pentylfuran, hexanal), fermentation (3-hydroxybutan-2-one, ethyl octanoate), and Maillard reactions (2,5-dimethylpyrazine, furan-2-carbaldehyde) [14]. The standard addition method was chosen to avoid potential matrix effects due to the different formulations of breads. Samples of 5 g each were prepared in a vial as described above with 0 µL, 10 µL, 100 µL, or 400 µL of a stock solution that contained the six compounds. The stock solution contained 5000 µg·g^−1^ 3-hydroxybutan-2-one (CAS number 513-86-0), 30 µg·g^−1^ hexanal (66-25-1), 10 µg·g^−1^ furan-2-carbaldehyde (98-01-1), 10 µg·g^−1^ 2,5-dimethylpyrazine (290-37-9), 10 µg·g^−1^ ethyl octanoate (106-32-1), and 5 µg·g^−1^ 2-pentylfuran (3777-69-3). Using the values generated by the four addition levels of the six compounds, a calibration curve (taking into account the dilution factor related to sample preparation) was created for each bread and each compound. Linear regression was then used to determine the concentration of each compound in each of the 11 breads.

### 2.6. Statistical Analysis

Statistical analysis was performed with XLSTAT software, v2016.1.1 (Addinsoft, Bordeaux, France). All statistical tests were performed on the mean values for each bread, with a cut-off at *p* = 0.05.

The normality of the variables was tested with a Shapiro–Wilk test, and homoscedasticity was tested using Levene’s test (Table S3). If the data were homoscedastic and followed a normal distribution, they were then analyzed using a one-way ANOVA (Analysis of Variance) on the ‘processing method’ factor, declined in three levels: ‘industrial’, ‘artisanal’, and ‘homemade’; followed by a post hoc Tukey HSD (Honestly Significant Difference) test to compare groups. If not, a Kruskal–Wallis test was used on the ‘processing method’ factor, followed by a Conover-Iman test [38]. In both cases, the Bonferroni correction was applied for multiple comparisons.

The experimentally derived nutritional data were compared to values present on the front-of-pack labeling using a Wilcoxon signed-rank test.

Correlations between variables were assessed using the Spearman method, as applied to non-normal data.

A correlation matrix Principal Component Analysis was performed using Pearson correlations and visualized as a distance biplot, with 95% confidence ellipses for each processing method. The same approach was taken for Multiple Factor Analysis, which was weighed by the groups of variables in each characterization (e.g., nutritional, chemical). Combined variables (e.g., the C* parameter calculated from a* and b*, or energy as the combination of macronutrients) were removed from the analysis. If a value was missing, it was replaced by the mean value of the variable.

## 3. Results and Discussion

### 3.1. Recipes and Technological Data

#### 3.1.1. Analysis of the Recipes

Recipes for industrial soft bread contained more ingredients than homemade or artisanal recipes (Table 2, *p* = 0.002). In particular, they typically contained numerous ingredients called adjuvants, whose role is to correct, improve, or facilitate bread production [39,40]. Among these, we identified wheat gluten (8/10 industrial soft breads), acerola extract (7/10), soybean flour (6/10), vinegar (5/10), and malted/fermented flours (2/10). Flavoring (either labeled as ‘natural’ or not) was also added to 8 of the 10 industrial breads, but was not found in any of the homemade or artisanal recipes in our selection. On the other hand, semi-skimmed milk was only found in homemade recipes (6/8), in which it was used to improve the softness of the final product. Butter was used in three of the four artisanal recipes and seven of the eight homemade recipes, but was not used in the industrial recipes, in which it was replaced mainly with vegetable oil (sunflower or rapeseed).

Similarly, the industrial recipes contained more additives than those intended for home use (*p* = 0.046 for the pairwise comparison). Only one artisanal recipe contained an antioxidant (in the flour), while the industrial recipes included up to seven additives, mainly texturants (7/15, including five emulsifiers and two hydrocolloids), preservatives (6/15), and antioxidants (2/15).

The proportion of fat in the recipes was lower for industrial breads compared to artisanal and homemade recipes (*p* = 0.004). Specifically, industrial soft breads contained more vegetable fat than the other preparations (Table 2, *p* = 0.005) but no animal fat (Table 2, *p* = 0.002). These differences in formulation are likely explained by the economic cost of products like butter and their logistical demands (i.e., cold storage, short shelf life). 

#### 3.1.2. Calculation of the Degree of Processing

The first step of this task was to create a production diagram for each soft bread. For homemade breads, the instructions in the recipes (i.e., dough preparation, time, and temperature for baking) were converted into a succession of unit operations; an example is given in Figure 1. We consulted professional bakers, academics, and experts on industrial baking in order to construct an accurate generic production diagram for artisanal soft bread and another for industrial soft bread.

Overall, the production diagrams of the three methods (displayed in Figure 1) are very similar, with major differences only in the production quantity and the equipment used. For example, dough division is not needed for homemade preparations because in most cases only a single loaf is prepared. For fermentation, a proofing cabinet or equivalent is available in the majority of industrial and artisanal facilities, but not in a home kitchen. The improved control of fermentation parameters in a professional setting allows the process to be accomplished more quickly and increases the level of standardization, which is essential for a commercial product.

Due to these similarities, Process-Scores were not significantly different among the three methods of production (Table 2, *p* = 0.287); they ranged from 40.49 to 41.82 for the four artisanal breads, from 40.22 to 45.70 for the 10 industrial breads, and from 36.37 to 45.45 for the eight homemade breads. These scores reflected the broad correspondence among the production steps of the different methods, and, as discussed in Section 3.1.1, the global similarity in recipe proportions (i.e., 60 ± 6 g of flour and 22 ± 8 g of water per 100 g of recipe).

As discussed above, the highest variability among recipes was found in the use of minority ingredients such as adjuvants and additives. This was reflected in the NOVA classification values, which were always highest (NOVA4) for the industrial soft breads (Table 1). All of the homemade and artisanal breads were classified as NOVA3, with the exception of P1 (artisanal), which was classified as NOVA4 because it contained an additive and ingredients such as powdered egg yolk. These differences in classification reflected the use of additives and/or characteristic substances such as wheat gluten, dextrose, or flavorings [41], which highlights that, here, the NOVA classification was more indicative of the recipe formulation than the processing method.

### 3.2. Nutritional Comparison

There were no differences among the three processing methods in terms of energy density (kcal·100 g^−1^) or carbohydrate, fiber, protein, and salt content (g·100 g^−1^) (Table 2, *p* = 0.501, 0.813, 0.523, 0.228, and 0.333, respectively).

**Figure 2:**
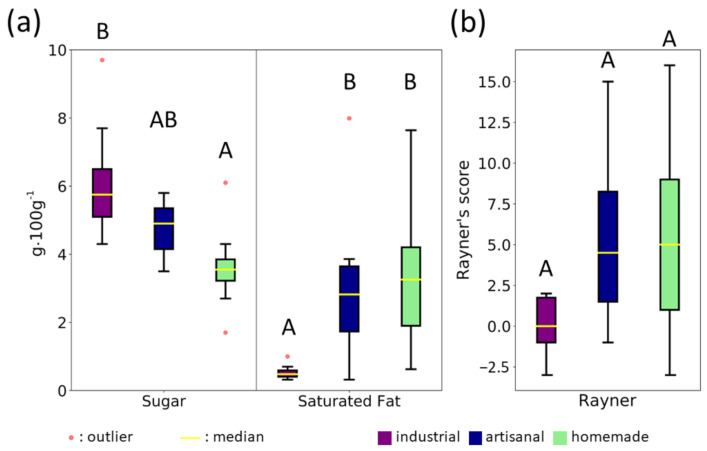
Nutritional comparison of the three processing methods. (**a**) Sugar and saturated fat content; (**b**) Rayner’s nutritional quality score. Data are represented by boxplots (1st and 3rd quartiles, median ± 1.5 × interquartile range for the whiskers), and statistically different groups are indicated with letters (according to post hoc Tukey or Conover-Iman tests, respectively).

Industrial soft breads had lower levels of saturated fatty acids than artisanal and homemade breads (Figure 2a and Table 2, *p* = 0.002). This is consistent with the recipe analysis in Section 3.1.1, which highlighted more vegetable fat and less animal fat in the industrial recipes compared to the other two methods (Table 1). However, the observed differences in the recipes did not translate into a statistically significant difference in overall fat content in the nutritional analysis (Table 2, *p* = 0.075). Finally, we found that industrial soft bread contained more sugar than homemade bread (Figure 2a and Table 2, *p* = 0.003).

With respect to Rayner’s score, no difference was detected in an overall analysis of the three processing methods (Table 2, *p* = 0.056). However, industrial soft bread did tend to have a lower Rayner’s score (0.0 ± 1.8) (Figure 2b).

It was not that surprising to find similar nutritional values among the breads examined here given the overall degree of similarity in the main ingredients in each recipe (Table 1). The subtle differences noted—such as in the use of butter and, to a lesser extent, milk—probably explain why the industrial soft bread contained less saturated fat. The higher sugar content of industrial bread could also be explained by the use of certain ingredients, notably soybean flour, which contains more sugar than wheat flour.

### 3.3. Physical Properties

#### 3.3.1. Comparison of the Density and Texture of Soft Bread

There were no significant differences among the three processing methods in terms of water content, density, or Young’s modulus (Table 2, *p* = 0.221, 0.100, and 0.329, respectively). Values of Young’s modulus were on the same order of magnitude as found in previous studies [42]. 

However, values of F_max_ and percentages of relaxation did differ among the three groups (Table 2, *p* = 0.020 and 0.040, respectively). This higher elasticity might be explained by the recipe formulation—for example, the use of texture additives such as emulsifiers (e.g., lactylates or mono- and diglycerides)—as well as better-controlled processing conditions that increase development of the bubble network. Additives such as reducing agents (e.g., ascorbic acid), and some enzymes categorized as processing aids (e.g., α-amylase) can also improve the elasticity of bread [43], and are sometimes found in industrial recipes.

#### 3.3.2. Color Comparison of Soft Breads

No significant differences were found among the three processing methods for the color parameters * and a* (Table 2, *p* = 0.577 and 0.426, respectively). For the b* parameter, and therefore the chromaticity C*, industrial soft bread had lower values than artisanal and homemade bread (Table 2, *p* = 0.013 and 0.014, respectively).

The yellow coloration of artisanal and homemade bread might be explained by differences in the recipes (Table 1), especially in the proportions of butter and egg (especially egg yolk) used. Indeed, the b* and C* parameters both appeared to be correlated with the percentage of butter in the recipe (*p* = 0.008 for both), with a Spearman correlation coefficient of 0.398.

### 3.4. Comparison of the Volatile Profiles of Soft Bread Crumb

Concentrations of the different markers were consistent with the existing literature [44,45,46,47]. Among the six molecules analyzed, differences in concentration among the three processing methods were noted only for ethyl octanoate, a marker of fermentation [14], which was less abundant in industrial compared to artisanal samples (Table 3, *p* = 0.024). This molecule could thus be used as an indicator for the discrimination of artisanal soft bread.

**Figure 3:**
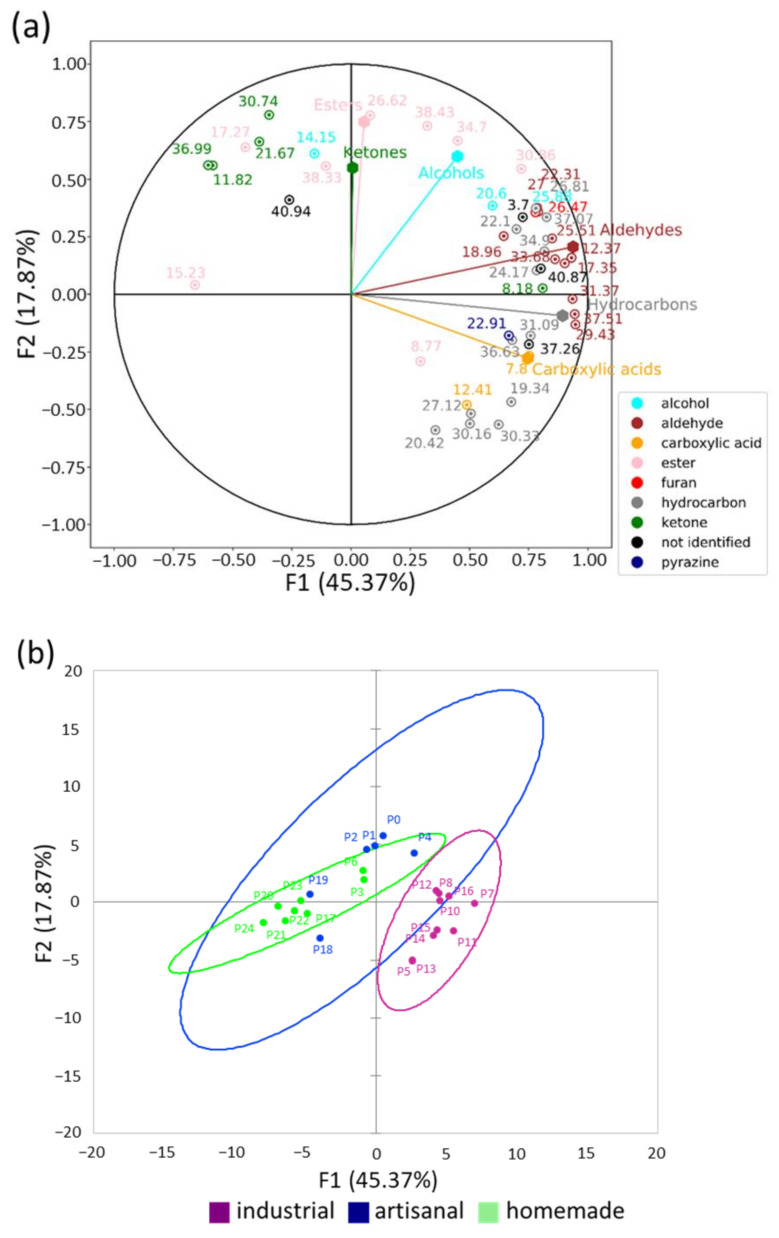
Representation of the correlations among the different volatile molecules in the 24 soft breads through a Principal Component Analysis (F1 + F2 = 63.24%) of areas under the curve. (**a**) Representation of 46 volatile molecules, separated into chemical families and labeled according to their mean retention time in Table 4; (**b**) biplot of the 24 soft breads, displayed by processing method, with 95% confidence ellipses.

Although these analyses did not highlight a strong impact of the processing methods on the selected markers, they did show that the matrix effect was relatively similar regardless of the bread under consideration; indeed, very similar slopes were observed for all calibration curves. We therefore chose to semi-quantitatively compare the areas under the curve for all the identified molecules (Table 4) to obtain a richer, more detailed characterization of the effects of the different processing methods on all 24 soft breads. Of the 81 volatile molecules analyzed, 46 demonstrated differences among the three production methods with respect to the areas under the curve (Table 4). We conducted a Principal Component Analysis using the concentrations of these 46 molecules (Figure 3) and found that, globally speaking, it was possible to discriminate between industrial and homemade soft bread. In contrast, the different examples of artisanal soft bread constituted a more heterogeneous group. When we repeated this process using all 81 molecules, the results were very similar (F1 + F2 = 49.25%, data not shown).

Only one molecule, nonanal, demonstrated clear differences among each of the three processing methods (Table 4, *p* = 0.0005). Because nonanal is related to lipid oxidation and the amount of yeast present [14], these results possibly reflect the higher amount of unsaturated fat (vegetable oils rather than butter) in industrial breads (Table 1 and Table 2) reported in Section 3.2. With respect to furans, industrial bread had higher concentrations of 2-pentylfuran (Table 4, *p* = 0.01) than homemade and artisanal bread, which could also be evidence of increased lipid oxidation in the industrial context [18]. The same trend was observed for most of the other aldehydes (e.g., pentanal, hexanal, benzaldehyde), which were all found in higher concentrations in industrial soft breads (Table 4 and Figure 3, *p* = 0.001, 0.001, and 0.015, respectively). These differences might be related to storage time [48], which is longer for industrial bread due to the inherent constraints of production, or to more intense kneading [49].

Another interesting result was found for propanoic acid (CAS number 79-09-4), which was not detected in artisanal and homemade soft bread but was clearly present in industrial samples that contained the E280 additive (Table 1 and Table 2, *p* = 0.005). The other carboxylic acid detected in the samples was acetic acid, which also appeared to be more concentrated in industrial bread (Table 2, *p* = 0.001); this was consistent with the fact that the industrial recipes were the only ones that included vinegar (Table 1). These compounds are involved in many pathways [14], but higher concentrations could reflect acidification related to strong fermentation activity, perhaps from the use of higher concentrations of yeast in order to reduce the time of production.

Certain esters (e.g., ethyl octanoate, ethyl butanoate) appeared to be more abundant in artisanal soft bread (Table 4 and Figure 3), which could also be reflective of more intense fermentation [46,50].

Products of the Maillard reactions, such as 2,5-dimethylpyrazine, furan-2-carbaldehyde, and 2-pentylfuran, were less abundant in homemade bread (Table 4, *p* = 0.003, 0.007, and 0.01, respectively). This would suggest that the Maillard reactions are less intense in homemade soft bread compared to the other methods, possibly due to a lower baking temperature, the type of sugar used, or even the lower amount of yeast, which would release fewer free amino acids than in other processing methods [50].

Generally, it was possible to differentiate between homemade and industrial soft bread on the basis of their profiles of volatile compounds, while artisanal and homemade bread were more similar overall (Figure 3b). There are several possible explanations for the observed differences. Fermentation appears to be longer and/or more intense in artisanal soft bread, while lipid oxidation seems to be more important in industrial bread, despite the presence of antioxidants and preservatives to lengthen shelf life. These differences may also translate into alterations in organoleptic perceptions [14]. Finally, the Maillard reactions seem to be more intense in industrial soft bread; this could increase concentrations of the carcinogens furan and/or 5-hydroxymethylfurfural, with potential consequences for health [17].

## 4. Conclusions

**Figure 4:**
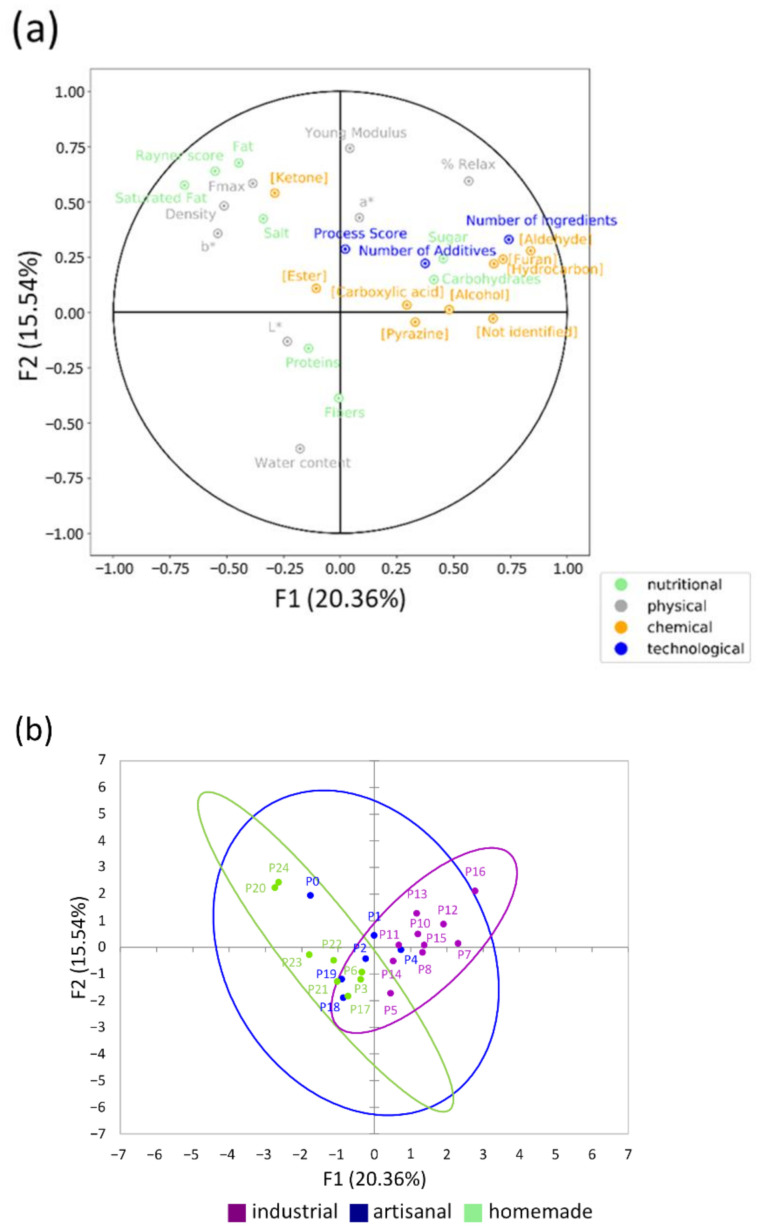
Multiple factorial analysis (F1 + F2 = 35.89%). (**a**) Representation of the correlations among the different variables quantified in each type of analysis (nutritional = rayner, total fat, saturated fat, total carbohydrate, sugar, fibers, proteins, salt; physical = density, F_max_, percentage of relaxation, Young’s modulus, L*, a*, b*, water content; chemical = 46 volatile molecules (Table 4), represented by their nine chemical families; technological = Process-Score, number of ingredients, number of additives, with the same weight for each of the four groups); (**b**) biplot of the 24 soft breads in this representation, presented according to processing method.

This work aimed to compare industrial, artisanal, and homemade methods of processing and characterize their impacts on the technological, nutritional, and physicochemical properties of soft bread (Figure 4). The experimental approach was deliberately designed to incorporate the realistic variability in products available to consumers. To this end, 24 different types of soft bread (10 industrial, 6 artisanal, and 8 homemade) were studied.

The main parameters supporting the differences between the different soft breads were the recipes (with characteristic ingredients of certain methods of processing), and therefore the contents of sugar and saturated fatty acids, as well as the chemical composition. Indeed, our analysis of bread crumb revealed higher concentrations of aldehydes in industrial bread, which hinted at the presence of more lipid oxidation. Esters were detected in higher concentrations in artisanal bread, which would suggest stronger fermentation. Finally, it seems that Maillard reactions might be less intense in homemade soft bread. The b* and C* color parameters and the elasticity also made it possible to highlight differences between the breads. However, the degree of processing, and other assessed nutritional and physical properties did not change according to the processing method.

The novelty of our approach is that it emphasizes the diversity within the category of soft bread. It would be interesting to use a similar approach on a larger sample set than the 24 breads examined here to see if it reinforces the trends we observed or reveals new differences.

In the future, the multicriteria mapping approach used here could be further enhanced with data on the sensory profiles of different soft breads, as well as with an investigation of contaminants (e.g., pesticides, mycotoxins, residual compounds coming from contact material), which can have important repercussions on health. Another interesting next step would be to study how differences between processing methods are perceived and addressed by consumers.

## Figures and Tables

**Table 1:** Detailed recipes for the soft breads of industrial, artisanal, and homemade origins used in this study. * refers to organic ingredients. Proportions are underlined when calculated with the Anatole^©^ software. The number of ingredients (including flavoring, if present), additives, and NOVA categories are indicated for each bread. NA: not applicable.

Soft Bread	Number of Ingredients (Including Flavoring)	Number of Additives	NOVA	Recipe: Ingredient and Quantity (% Total Ingredients)	
P0 (artisanal)	7 (0)	0	3	flour	55.6
				water	13.6
				butter	12.5
				eggs	10.1
				sugar	3.9
				yeast	3.0
				salt	1.3
P1 (artisanal)	11 (0)	1	4	flour (wheat flour, wheat gluten, maltead wheat flour)	59.2
				water	30.3
				butter	4.6
				sugar	4.5
				salt	1.0
				egg yolk powder	0.4
				yeast	
				deactivated yeast	
				flour treatment agent E300	
P2 (artisanal)	NA	NA	NA	not available	
P3 (homemade)	8 (0)	0	3	T45 flour	57.5
				water	30.0
				sugar	2.9
				eggs	2.9
				butter	2.3
				sunflower oil	1.7
				yeast	1.7
				salt	1.0
P4 (artisanal)	NA	NA	NA	not available	
P5 (industrial)	8 (1)	0	4	wheat flour	73.0
				water	20.8
				rapeseed oil	2.2
				yeast	2.2
				salt	1.0
				flavor (contains alcohol)	0.5
				fermented corn flour	
				acerola extract	
P6 (homemade)	8 (0)	0	3	T45 flour	57.5
				water	30.0
				sugar	2.9
				eggs	2.9
				butter	2.3
				sunflower oil	1.7
				yeast	1.7
				salt	1.0
P7 (industrial)	10 (1)	0	4	T80 wheat flour	63
				water	25.8
				cane sugar	4.8
				sunflower oil	3.0
				yeast	1.9
				salt	1.0
				vinegar	0.5
				wheat gluten	
				natural flavor (contains alcohol)	
				acerola extract	
P8 (industrial)	11 (1)	0	4	wheat flour	63.0
				water	23.7
				sugar	4.6
				rapeseed oil	3.2
				bean flour	2.0
				yeast	2.0
				salt	1.0
				vinegar	0.2
				wheat gluten	
				flavor (contains alcohol)	
				acerola extract	
P10 (industrial)	12 (1)	1	4	wheat flour	66.5
				water	21.9
				rapeseed oil	3.1
				sugar	2.9
				yeast	2.0
				bean flour	2.0
				salt	1.1
				wheat gluten	0.2
				vinegar	0.1
				flavor (contains alcohol)	
				preservative: calcium propionate	
				acerola extract	
P11 (industrial)	9 (1)	0	4	wheat flour	70.0
				water	16.9
				rapeseed oil	4.0
				yeast	4.0
				bean flour	2.0
				vinegar	1.7
				salt	1.0
				flavor (contains alcohol)	
				acerola extract	
P12 (industrial)	17 (1)	7	4	wheat flour (contains gluten)	57.0
				water	30.0
				sugar	2.7
				sunflower vegetable oil	2.7
				yeast	1.8
				wheat gluten	1.7
				soybean flour	1.2
				dextrose	0.9
				salt	0.8
				emulsifiers: sodium stearoyl-2-lactylate, mono- and diglycerides of fatty acids	
				preservatives: calcium propionate, sorbic acid, potassium sorbate	
				stabilizer: guar gum	
				flour treatment agent: ascorbic acid	
				flavor (contains alcohol)	
P13 (industrial)	13 (1)	4	4	wheat flour	67.0
				water	20.7
				sugar	5.8
				rapeseed oil	2.0
				wheat gluten	1.7
				yeast	1.2
				salt	0.9
				flavor	0.3
				preservative: calcium propionate	
				emulsifiers: mono- and diglycerides of fatty acids, lecithin	
				thickener: xanthan gum	
				bean flour	
P14 (industrial)	12 (0)	2	4	wheat flour	63.0
				water	29.6
				yeast	4.7
				wheat fiber	1.3
				salt	1.0
				vinegar	0.3
				wheat gluten	
				bean flour	
				emulsifier: mono- and diglycerides of fatty acids (rapeseed)	
				preservative: calcium propionate	
				psyllium	
				acerola extract	
P15 (industrial)	8 (1)	0	4	wheat flour *	65.0
				water	25.4
				rapeseed oil *	3.9
				cane sugar *	3.3
				salt	1.0
				yeast	1.0
				wheat gluten *	0.4
				natural flavor * (contains alcohol *)	
P16 (industrial)	13 (0)	0	4	wheat flour *	47
				water	29.8
				sourdough* (wheat flour *, water, yeast, untreated sea salt)	14.5
				sunflower oil *	3.0
				white cane sugar *	3.0
				wheat gluten *	1.8
				malted wheat flour *	0.7
				yeast *	0.2
				untreated sea salt	
				acerola extract	
P17 (homemade)	7 (0)	0	3	T45 flour	55.5
				water	18.6
				milk	18.5
				oil	3.9
				sugar	1.8
				baker’s yeast	1.1
				salt	0.6
P18 (artisanal)	5 (0)	0	3	traditional flour	65.9
				water	30.9
				sourdough	2.0
				salt	1.1
				baker’s yeast	0.1
P19 (artisanal)	6 (0)	0	3	flour	55.6
				water	30.4
				butter	5.6
				sugar	5.6
				yeast	1.7
				salt	1.1
P20 (homemade)	7 (0)	0	3	flour	66.5
				water	15.0
				soft butter	8.7
				semi-skimmed milk	4.2
				baker’s yeast	2.8
				sugar	1.4
				salt	1.4
P21 (homemade)	8 (0)	0	3	flour	59.5
				milk	18.1
				water	12.1
				butter	4.1
				fresh yeast	2.0
				sugar	2.0
				eggs	1.2
				salt	1.0
P22 (homemade)	7 (0)	0	3	flour	54.6
				milk	21.8
				water	10.9
				butter	5.6
				sugar	4.4
				fresh yeast	2.2
				salt	0.5
P23 (homemade)	7 (0)	0	3	flour	51.5
				milk	25.7
				water	12.5
				butter	5.3
				fresh yeast	2.0
				sugar	1.5
				salt	1.5
P24 (homemade)	6 (0)	0	3	flour	56.3
				semi-skimmed milk	33.8
				sweet butter	6.9
				sugar	1.1
				salt	1.1
				baker’s yeast	0.8

**Table 2:** Statistical comparison of the different tested variables (units in parentheses) among the three processing methods. Mean values ± standard deviation (n = values taken into account, if different from the group as a whole) are shown for each processing method, with the *p* value of an ANOVA or Kruskal–Wallis test. Statistical groups determined by post hoc test (Tukey or Conover-Iman, respectively) are indicated by letters. * for *p* ≤ 0.05, and ** for *p* ≤ 0.01, in **bold** when below the threshold of 0.05.

	Variable		Industrial (n = 10)		Artisanal (n = 6)		Homemade (n = 8)		*p* Value
R E C I P E	Number of ingredients		11.3 ± 2.8	B	7 ± 2.8 (n = 4)	A	7.23 ± 0.7	A	0.002 **
	Number of additives		1.4 ± 2.4	A	0.3 ± 0.5 (n = 4)	A	0.0 ± 0.0	A	0.132
	Process-Score		43.0 ± 1.8	A	41.1 ± 0.6 (n = 4)	A	41.4 ± 3.4	A	0.287
	% animal fat		0.0 ± 0.0	A	6.7 ± 6.8 (n = 4)	B	4.7 ± 2.6	B	0.002 **
	% vegetable fat		2.7 ± 1.1	B	0.0 ± 0.0 (n = 4)	A	0.9 ± 1.4	A	0.005 **
P H Y S I C A L	Water content (g·100 g^−1^)		38.4 ± 3.6	A	41.9 ± 3.0	A	39.7 ± 2.1	A	0.221
	Density		0.2 ± 0.0 (n = 5)	A	0.2 ± 0.1	A	0.3 ± 0.1	A	0.100
	F_max_ (N)		1.6 ± 0.8	AB	1.2 ± 0.5	A	3.1 ± 2.7	B	0.020 *
	Relaxation (%)		36.4 ± 5.0	B	32.1 ± 3.7	AB	30.9 ± 4.0	A	0.040 *
	Young’s modulus (kPa)		9.3 ± 9.1	A	4.7 ± 3.7	A	9.3 ± 7.8	A	0.329
	L*		73.7 ± 3.8 (n = 5)	A	72.8 ± 2.0	A	74.4 ± 2.1	A	0.577
	a*		0.5 ± 0.8 (n = 5)	A	0.4 ± 0.3	A	0.1 ± 0.5	A	0.426
	b*		14.6 ± 1.3 (n = 5)	A	19.3 ± 3.8	B	19.4 ± 2.3	B	0.013 *
	C*		14.6 ± 1.3 (n = 5)	A	19.3 ± 3.8	B	19.4 ± 2.3	B	0.014 *
N U T R I T I O N A L	Energy (kcal·100 g^−1^)		270.0 ± 15.4	A	280.8 ± 33.3	A	285.5 ± 21.9	A	0.501
	Total fat	(g· 100 g^−1^)	4.0 ± 1.3	A	5.6 ± 4.1	A	6.3 ± 2.2	A	0.075
	Saturated Fat		0.5 ± 0.2	A	3.2 ± 2.7	B	3.5 ± 2.2	B	0.002 **
	Total Carbohydrate		50.5 ± 3.2	A	51.2 ± 1.9	A	51.8 ± 1.2	A	0.813
	Sugars		6.1 ± 1.6	B	4.8 ± 0.9	AB	3.6 ± 1.3	A	0.003 **
	Fibers		3.4 ± 1.0	A	2.9 ± 0.8	A	3.2 ± 0.4	A	0.523
	Proteins		8.2 ± 0.9	A	8.6 ± 1.2	A	7.8 ± 0.3	A	0.228
	Salt		1.2 ± 0.2	A	1.3 ± 0.1	A	1.3 ± 0.4	A	0.333
	Rayner’s score		0.0 ± 1.8	A	5.5 ± 5.9	A	5.4 ± 6.2	A	0.056

**Table 3:** Comparison of the six volatile molecules tested with the standard addition method (concentration in µg·kg^−1^) among the three processing methods. Mean values ± standard deviation are shown for each method, with the *p* value of an ANOVA or Kruskal–Wallis test. Statistical groups determined by post hoc test (Tukey or Conover-Iman, respectively) are indicated by letters. * for *p* ≤ 0.05, in **bold** when below the threshold of 0.05.

Molecule	Industrial (n = 5)		Artisanal (n = 4)		Homemade (n = 2)		*p* Value
[3-hydroxybutan-2-one]	66,667.0 ± 36,947.9	A	112,252.7 ± 47,334.5	A	90,060.7 ± 20,794.9	A	0.286
[hexanal]	935.1 ± 522.7	A	450.3 ± 98.4	A	523.8 ± 102.2	A	0.178
[furan-2-carbaldehyde]	128.3 ± 129.4	A	191.7 ± 270.7	A	26.5 ± 2.9	A	0.767
[2,5-dimethylpyrazine]	0.8 ± 1.8	A	0.0 ± 0.0	A	15.8 ± 22.3	A	0.301
[2-pentylfuran]	1581.9 ± 1173.6	A	597.1 ± 450.0	A	831.7 ± 196.2	A	0.313
[ethyl octanoate]	193.7 ± 108.9	A	1694.1 ± 1417.8	B	251.9 ± 15.9	AB	0.023 *

**Table 4:** Areas under the curve of the intensity–retention time plot ((mean ± standard deviation) × 10^3^) for the 46 volatile molecules that demonstrated significant differences among processing methods. Compounds are displayed by their IUPAC (International Union of Pure and Applied Chemistry) names and chemical classifications. Kovats retention indexes were taken from PubChem and consolidated with data from the National Institute of Standards and Technology database for a semi-standard non-polar column. n is the total number of soft breads sampled. Molecules that are underlined were treated by EIC; all others were treated by TIC. Mean values ± standard deviation are shown for each method, with the *p* value of an ANOVA or Kruskal–Wallis test. Statistical groups determined by post hoc test (Tukey or Conover-Iman, respectively) are indicated by letters. * for *p* ≤ 0.05, ** for *p* ≤ 0.01, and *** for *p* ≤ 0.001, in **bold** when below the threshold of 0.05. NA: not applicable.

Compound	CAS Number	Chemical Classification	Kovats Retention Index	Retention Time (min)	Industrial (n = 10)		Artisanal (n = 6)		Homemade (n = 8)		*p* Value
Not identified	NA	NA	NA	3.70 ± 0.05	17,271.0 ± 4088.3	B	13,682.6 ± 6470.0	AB	9075.4 ± 4849.0	A	0.009 **
acetic acid	64-19-7	Carboxylic acid	619 ± 22	7.80 ± 0.14	465.0 ± 644.7	B	22.0 ± 50.7	A	0.1 ± 0.0	A	0.001 ***
butan-2-one	78-93-3	Ketone	587 ± 23	8.18 ± 0.07	68.6 ± 31.0	B	42.4 ± 36.2	AB	17.9 ± 14.4	A	0.004 **
ethyl acetate	141-78-6	Ester	609 ± 13	8.77 ± 0.06	3809.0 ± 4 835.0	B	493.5 ± 375.8	A	1549.3 ± 1284.3	AB	0.050 *
pentan-2-one	107-87-9	Ketone	679 ± 22	11.82 ± 0.06	5.2 ± 8.0	A	91.9 ± 161.6	B	84.2 ± 74.5	B	0.001 ***
pentanal	110-62-3	Aldehyde	698 ± 14	12.37 ± 0.05	281.9 ± 204.8	B	108.9 ± 69.7	A	46.2 ± 40.5	A	0.001 ***
propanoic acid	79-09-4	Carboxylic acid	704 ± 24	12.41 ± 0.64	2523 ± 4 230.1	B	0.1 ± 0.0	A	0.1 ± 0.0	A	0.005 **
3-methylbutan-1-ol	123-51-3	Alcohol	738 ± 11	14.15 ± 0.08	10,972.8 ± 6670.7	A	18,993.2 ± 7 235.3	A	21,881.2 ± 11,862.3	A	0.043 *
ethyl 2-methylpropanoate	97-62-1	Ester	755 ± 10	15.23 ± 0.05	1.5 ± 1.4	A	6.6 ± 12.6	A	54.3 ± 53.5	B	0.002 **
ethyl butanoate	105-54-4	Ester	798 ± 10	17.27 ± 0.04	3.9 ± 1.6	A	48.3 ± 56.8	B	24.6 ± 12.7	B	0.003 **
hexanal	66-25-1	Aldehyde	797 ± 34	17.35 ± 0.05	918.4 ± 489.1	B	375.6 ± 235.7	A	184 ± 180.9	A	0.001 ***
furan-2-carbaldehyde	98-01-1	Aldehyde	830 ± 53	18.96 ± 0.07	197.1 ± 213.6	B	277.3 ± 506.4	B	12.1 ± 21.6	A	0.007 **
2,4-dimethylhept-1-ene	19549-87-2	Hydrocarbon	838 ± 10	19.34 ± 0.02	298.1 ± 339.7	B	1.4 ± 0.9	A	1.2 ± 0.3	A	0.002 **
4-methyloctane	2216-34-4	Hydrocarbon	863 ± 3	20.42 ± 0.03	33.4 ± 38.0	B	1.7 ± 1.7	A	2.0 ± 1.3	AB	0.028 *
hexan-1-ol	111-27-3	Alcohol	865 ± 50	20.60 ± 0.06	823.6 ± 563.0	B	779.0 ± 341.5	B	299.4 ± 221.7	A	0.021 *
heptan-2-one	110-43-0	Ketone	888 ± 11	21.67 ± 0.05	126.7 ± 71.4	A	868.5 ± 1084.4	AB	658.3 ± 487.2	B	0.021 *
styrene	100-42-5	Hydrocarbon	837 ± 197	22.10 ± 0.05	122.6 ± 80.7	B	80.1 ± 58.1	AB	51.0 ± 44.1	A	0.039 *
heptanal	111-71-7	Aldehyde	897 ± 46	22.31 ± 0.04	490.9 ± 321.5	B	443.6 ± 357.1	AB	162.8 ± 129.5	A	0.009 **
2,5-dimethylpyrazine	123-32-0	Pyrazine	916 ± 12	22.91 ± 0.05	11.3 ± 13.1	B	3.1 ± 1.3	AB	1.9 ± 2.8	A	0.003 **
2,6,6-trimethylbicyclo[3.1.1]hept-2-ene	80-56-8	Hydrocarbon	936 ± 8	24.17 ± 0.02	218.8 ± 247.9	B	33.0 ± 46.9	AB	3.4 ± 3.9	A	0.002 **
benzaldehyde	100-52-7	Aldehyde	954 ± 80	25.51 ± 0.06	435.8 ± 181.0	B	229.8 ± 203.9	AB	151.1 ± 209.3	A	0.015 *
oct-1-en-3-ol	3391-86-4	Alcohol	980 ± 7	25.88 ± 0.04	74.2 ± 110.5	B	34.0 ± 20.6	AB	22.5 ± 38.3	A	0.035 *
2-pentylfuran	3777-69-3	Furan	992 ± 6	26.47 ± 0.03	546.1 ± 277.5	B	378.3 ± 141.5	AB	174.8 ± 208.8	A	0.010 **
ethyl hexanoate	123-66-0	Ester	994 ± 67	26.62 ± 0.04	136.6 ± 71.9	A	758.3 ± 595.2	B	165.5 ± 106.1	AB	0.043 *
decane	124-18-5	Hydrocarbon	1000	26.81 ± 0.02	294.4 ± 763.9	B	73.0 ± 112.4	AB	16.6 ± 11.6	A	0.014 *
octanal	124-13-0	Aldehyde	998 ± 63	27.00 ± 0.04	118.3 ± 68.5	B	66.2 ± 74.4	AB	20.5 ± 29.1	A	0.03 *
2,6-dimethylnonane	17302-28-2	Hydrocarbon	1020 ± 4	27.12 ± 0.11	82.4 ± 99.9	B	1.0 ± 0.0	A	1.0 ± 0.0	A	0.016 *
(E)-oct-2-enal	2548-87-0	Aldehyde	1059 ± 8	29.43 ± 0.01	151.2 ± 138.8	B	15.4 ± 17.4	A	8.3 ± 13.7	A	0.0001 ***
Not identified	NA	Hydrocarbon	NA	30.16 ± 0.02	145.1 ± 152.1	B	1.6 ± 0.9	A	2.2 ± 2.4	A	0.007 **
Not identified	NA	Hydrocarbon	NA	30.33 ± 0.01	121.5 ± 124.3	B	1.0 ± 0.0	A	1.0 ± 0.0	A	0.002 **
nonan-2-one	821-55-6	Ketone	1085 ± 73	30.74 ± 0.03	25.0 ± 35.2	A	245.3 ± 245.6	B	116.0 ± 79.9	B	0.012 *
ethyl heptanoate	106-30-9	Ester	1095 ± 9	30.86 ± 0.02	9.0 ± 7.2	B	19.5 ± 20.4	A	1.3 ± 1.4	A	0.007 **
Not identified	NA	Hydrocarbon	NA	31.09 ± 0.01	746.2 ± 1 744.6	B	16.0 ± 11.9	A	11.8 ± 13.9	A	0.005 **
nonanal	124-19-6	Aldehyde	1101 ± 40	31.37 ± 0.04	401.6 ± 137.3	C	199.1 ± 151.7	B	71.9 ± 96.5	A	0.0005 ***
(E)-non-2-enal	18829-56-6	Aldehyde	1162 ± 7	33.68 ± 0.03	74.9 ± 27.5	B	48.2 ± 46.8	B	7.6 ± 13.2	A	0.001 ***
ethyl octanoate	106-32-1	Ester	1188 ± 93	34.70 ± 0.02	318.5 ± 156.7	AB	1989.5 ± 1 875.4	B	135.5 ± 123.7	A	0.014 *
dodecane	112-40-3	Hydrocarbon	1200	34.90 ± 0.01	414.5 ± 1 129.5	B	33.6 ± 44.2	AB	10.7 ± 7.1	A	0.005 **
Not identified	NA	Hydrocarbon	NA	36.63 ± 0.01	42.2 ± 46.9	B	4.1 ± 4.2	AB	3.4 ± 5.3	A	0.020 *
undecan-2-one	112-12-9	Ketone	1286 ± 84	36.99 ± 0.03	1.1 ± 0.2	A	59.2 ± 76.6	B	14.2 ± 13.6	B	0.002 **
tridecane	629-50-5	Hydrocarbon	1300	37.07 ± 0.01	172.8 ± 312.4	B	35.0 ± 30.9	AB	14.6 ± 24.5	A	0.013 *
Not identified	NA	NA	NA	37.26 ± 0.01	60.7 ± 49.7	B	12.3 ± 8.8	AB	7.8 ± 11.2	A	0.006 **
(2E,4E)-deca-2,4-dienal	25152-84-5	Aldehyde	1318 ± 10	37.51 ± 0.02	256.8 ± 267.5	B	21.2 ± 10.4	A	14.7 ± 21.1	A	0.0001 ***
ethyl dec-9-enoate	67233-91-4	Ester	1387 ± 2	38.33 ± 0.02	1.2 ± 0.7	A	72.5 ± 60.3	B	1.8 ± 1.8	A	0.004 **
ethyl decanoate	110-38-3	Ester	1378 ± 141	38.43 ± 0.02	25.7 ± 21.3	AB	232.8 ± 259.2	B	15.8 ± 12.6	A	0.041 *
Not identified	NA	NA	NA	40.87 ± 0.02	67.7 ± 16.1	B	44.2 ± 41.0	AB	13.5 ± 23.4	A	0.004 **
Not identified	NA	NA	NA	40.94 ± 0.00	368.0 ± 134.2	A	604.9 ± 200.5	B	463 ± 152.9	AB	0.025 *

## Data Availability

The original contributions presented in the study are included in the article/supplementary material; further inquiries can be directed to the corresponding author.

## Supplementary Materials

The following supporting information can be downloaded at: https://www.mdpi.com/article/10.3390/foods11101484/s1. Table S1: Detailed data relating to the Process-Score of soft breads. (a) Extract from the catalog of generic unit operations used for soft bread production diagrams; (b) Process-Scores of the main soft bread ingredients. Table S2: Selected ions corresponding to the chromatographic peaks of the volatile compounds treated by extracted ion chromatogram analysis. Table S3: Normality and homoscedasticity testing of the variables.
