# Supplementary material for: How Different Are Industrial, Artisanal and Homemade Soft Breads?
Source: Foods. 2022 May 19;11(10):1484. doi: 10.3390/foods11101484 (PMC9140824; doi:10.3390/foods11101484)
Supplement: Supplementary file 1 [file foods-11-01484-s001.zip › foods-1711810-Supplementary.pdf]

foods

Article

Supplementary Data—How Different Are Industrial, Artisanal, and Homemade Soft Breads?

Bastien Maurice[1], Anne Saint-Eve[1], Aurélia Pernin[1], Pascal Leroy[2] and Isabelle Souchon[3,*]

[1] Université Paris-Saclay, INRAE, AgroParisTech, UMR SayFood, F-91120 Palaiseau, France
[2] Université Paris-Saclay, INRAE, UMR PSAE, F-91120 Palaiseau, France
[3] INRAE, Avignon Université, UMR SQPOV, F-84000 Avignon, France
* Correspondence: isabelle.souchon@inrae.fr

Table S1. Detailed data relating to the Process-Score of soft breads: (**a**) Extract from the catalog of generic unit operations used for soft bread production diagrams; (**b**) Process-Scores of the main soft bread ingredients.

(**a**)

Unit operation	Category	Description	Points
Cutting	Mechanical	Simple, relatively rough cutting	0.5
Homogenous mixture	Mechanical	Mixing 2 or more compounds in the same state (gas, liquid, or solid)	0.5
Solid-liquid mixture	Mechanical	Mixing 2 or more compounds in different states	1
Crushing / slow compression	Mechanical	Flattening something by strong compression	1.5
Kneading	Mechanical	Pressing multiple times to give shape and structure	2
Cooling	Thermal	Reducing the temperature of the product up to 0–4 °C	0.5
Liquid bleeding	Thermal	Loss of liquid during a period of cooling, drying, or relaxation	1
Tempering (proofing)	Thermal	Tempering in a humid atmosphere, in a closed area (< 40 °C)	3
Medium heating	Thermal	Oven heating, in the 100–150 °C range for < 30 min	6
Intense heating	Thermal	More intense heating, in the 150–200 °C range for < 30 min ∣ or 100-150 °C for > 30 min	7.5
Very intense heating	Thermal	Very strong heating, close to roasting, with temperatures > 200 °C ∣ or 150-200 °C for > 30 min	9
Short yeast fermentation	Biological	Short microbiological action (< 1 day)	4

(**b**)

Ingredient	Total Process-Score
Water	0.00
Butter	14.00
Wheat flour (T80)	22.00
Sugar	23.00
Wheat flour (T45)	26.50
Salt	31.00
Fresh yeast	33.25
Dry yeast	39.25
Colza oil / Sunflower oil	40.50

Table S2: Selected ions corresponding to the chromatographic peaks of the volatile compounds treated by extracted ion chromatogram

Compound	CAS Number	Kovats Retention Index	Retention time (min)	Selected ions
acetic acid	64-19-7	619 ± 22	7.80 ± 0.14	45; 60
pentanal	110-62-3	698 ± 14	12.37 ± 0.05	39; 41; 44; 58
hexanal	66-25-1	797 ± 34	17.35 ± 0.05	56; 82
2,5-dimethylpyrazine	123-32-0	916 ± 12	22.91 ± 0.05	42; 81; 108
ethyl butanoate	105-54-4	798 ± 10	17.27 ± 0.04	88
butan-2-one	78-93-3	587 ± 23	8.18 ± 0.07	72
propanoic acid	79-09-4	704 ± 24	12.41 ± 0.64	73; 74
ethyl heptanoate	106-30-9	1,095 ± 9	30.86 ± 0.02	60; 61; 73; 88; 101; 113; 115
benzaldehyde	100-52-7	954 ± 80	25.51 ± 0.06	77; 105; 106
2-phenylethanol	60-12-8	1,112 ± 58	32.05 ± 0.07	92; 122
butane-2,3-dione	431-03-8	596 ± 12	7.98 ± 0.06	86
3-hydroxybutan-2-one	513-86-0	713 ± 13	12.97 ± 0.08	43; 88
2-methylpropanal	78-84-2	552 ± 12	7.12 ± 0.06	72
propan-1-ol	71-23-8	551 ± 19	7.03 ± 0.08	31; 59; 60

Table S3. Normality and homoscedasticity testing of the variables. * for $p \leq 0.05$, ** for $p \leq 0.01$, and *** for $p \leq 0.001$, in **bold** when below the threshold of 0.05.

	Variable	Total number of values n (industrial/artisanal/homemade)	*p* value (Shapiro-Wilk test)	*p* value (Levene test)	Selected test
RECIPE	Number of ingredients	n = 22 (10/4/8)	0.058	0.424	ANOVA
	Number of additives		0.002**	0.020*	Kruskal-Wallis
	Process-Score		< 0.0001***	< 0.0001***	Kruskal-Wallis
	% animal fat		< 0.0001***	0.002**	Kruskal-Wallis
	% vegetable fat		0.001***	0.051	Kruskal-Wallis
PHYSICAL	Water content	n = 24 (10/6/8)	0.064	0.027*	ANOVA
	F_{max} (N)		< 0.0001***	0.006**	Kruskal-Wallis
	Relaxation (%)		0.356	0.281	ANOVA
	Young's modulus (kPa)		0.018*	0.186	Kruskal-Wallis
	Density	n = 19 (5/8/6)	< 0.0001***	0.005**	Kruskal-Wallis
	L*		0.023*	0.720	ANOVA
	a*		0.012*	0.016*	Kruskal-Wallis
	b*		0.306	0.387	ANOVA
	C*		0.017*	0.004**	ANOVA
CHEMICAL	[3-hydroxybutan-2-one]	n = 11 (5/4/2)	0.445	0.525	ANOVA
	[hexanal]		0.003**	0.006**	Kruskal-Wallis
	[furan-2-carbaldehyde]		0.004**	0.172	Kruskal-Wallis
	[2,5-dimethylpyrazine]		< 0.0001***	< 0.0001***	Kruskal-Wallis
	[2-pentylfuran]		0.02*	0.258	Kruskal-Wallis
	[ethyl octanoate]		< 0.0001***	0.019*	Kruskal-Wallis
NUTRITIONAL	Energy	n = 24 (10/6/8)	0.074	0.106	Kruskal-Wallis
	Fats		< 0.0001***	< 0.0001***	Kruskal-Wallis
	Saturated Fatty Acids		0.0002***	0.724	Kruskal-Wallis
	Carbohydrates		< 0.0001***	0.006**	Kruskal-Wallis
	Sugar		0.003**	0.091	ANOVA
	Fibers		< 0.0001***	0.016*	Kruskal-Wallis
	Proteins		< 0.0001***	0.002**	Kruskal-Wallis
	Salt		< 0.0001***	< 0.0001***	Kruskal-Wallis
	Rayner's score		< 0.0001***	0.001***	Kruskal-Wallis
GC-MS	Not identified (3.7)	n = 24 (10/6/8)	< 0.0001***	0.001***	ANOVA
	acetic acid (7.8)		< 0.0001***	0.001***	Kruskal-Wallis
	butan-2-one (8.18)		0.001***	0.369	Kruskal-Wallis
	ethyl acetate (8.77)		< 0.0001***	0.036*	Kruskal-Wallis
	pentan-2-one (11.82)		< 0.0001***	< 0.0001***	Kruskal-Wallis
	pentanal (12.37)		< 0.0001***	0.078	Kruskal-Wallis
	propanoic acid (12.41)		< 0.0001***	0.002**	Kruskal-Wallis
	3-methylbutan-1-ol (14.15)		0.008**	0.586	ANOVA
	ethyl 2-methylpropanoate (15.23)		< 0.0001***	0.0003***	Kruskal-Wallis

ethyl butanoate (17.27)		**< 0.0001*** **	**< 0.0001*** **	Kruskal-Wallis
hexanal (17.35)		**< 0.0001*** **	**< 0.0001*** **	Kruskal-Wallis
furan-2-carbaldehyde (18.96)		**< 0.0001*** **	**0.001*** **	Kruskal-Wallis
2,4-dimethylhept-1-ene (19.34)		**< 0.0001*** **	0.051	Kruskal-Wallis
4-methyloctane (20.42)		**< 0.0001*** **	**< 0.0001*** **	Kruskal-Wallis
hexan-1-ol (20.6)		**< 0.0001*** **	0.059	Kruskal-Wallis
heptan-2-one (21.67)		**< 0.0001*** **	**0.001*** **	Kruskal-Wallis
styrene (22.1)		**0.003** **	0.084	Kruskal-Wallis
heptanal (22.31)		**< 0.0001*** **	**< 0.0001*** **	Kruskal-Wallis
2,5-dimethylpyrazine (22.91)		**< 0.0001*** **	**< 0.0001*** **	Kruskal-Wallis
2,6,6-trimethylbicyclo[3.1.1]hept-2-ene (24.17)		**< 0.0001*** **	**0.045* **	Kruskal-Wallis
benzaldehyde (25.51)		**< 0.0001*** **	**< 0.0001*** **	Kruskal-Wallis
oct-1-en-3-ol (25.88)		**< 0.0001*** **	**0.002** **	Kruskal-Wallis
2-pentylfuran (26.47)		**< 0.0001*** **	**0.011* **	ANOVA
ethyl hexanoate (26.62)		**< 0.0001*** **	0.200	Kruskal-Wallis
decane (26.81)		**< 0.0001*** **	**< 0.0001*** **	Kruskal-Wallis
octanal (27)		0.125	**0.025* **	Kruskal-Wallis
2,6-dimethylnonane (27.12)		0.075	0.119	Kruskal-Wallis
(E)-oct-2-enal (29.43)		**0.005** **	0.395	Kruskal-Wallis
Not identified (30.16)		**0.0001*** **	0.082	Kruskal-Wallis
Not identified (30.33)		0.058	0.424	Kruskal-Wallis
nonan-2-one (30.74)		**0.002** **	**0.020* **	Kruskal-Wallis
ethyl heptanoate (30.86)		0.064	**0.027* **	Kruskal-Wallis
Not identified (31.09)		**< 0.0001*** **	**0.006** **	Kruskal-Wallis
nonanal (31.37)		0.356	0.281	Kruskal-Wallis
(E)-non-2-enal (33.68)		**< 0.0001*** **	**0.005** **	Kruskal-Wallis
ethyl octanoate (34.7)		**0.023* **	0.720	Kruskal-Wallis
dodecane (34.9)		**0.012* **	**0.016* **	Kruskal-Wallis
Not identified (36.63)		**0.017* **	**0.004** **	Kruskal-Wallis
undecan-2-one (36.99)		**< 0.0001*** **	**0.002** **	Kruskal-Wallis
tridecane (37.07)		**0.04* **	0.052	Kruskal-Wallis
Not identified (37.26)		0.498	0.100	Kruskal-Wallis
(2E,4E)-deca-2,4-dienal (37.51)		0.074	0.106	Kruskal-Wallis
ethyl dec-9-enoate (38.33)		**0.0002*** **	0.724	Kruskal-Wallis
ethyl decanoate (38.43)		**< 0.0001*** **	**0.006** **	Kruskal-Wallis
Not identified (40.87)		**< 0.0001*** **	**< 0.0001*** **	Kruskal-Wallis
Not identified (40.94)		**< 0.0001*** **	**0.001*** **	Kruskal-Wallis
